# Metabolomic signatures of low birthweight: Pathways to insulin resistance and oxidative stress

**DOI:** 10.1371/journal.pone.0194316

**Published:** 2018-03-22

**Authors:** Sarah Jane Metrustry, Ville Karhunen, Mark H. Edwards, Cristina Menni, Thomas Geisendorfer, Anja Huber, Christian Reichel, Elaine M. Dennison, Cyrus Cooper, Tim Spector, Marjo-Riitta Jarvelin, Ana M. Valdes

**Affiliations:** 1 Department of Twin Research and Genetic Epidemiology, Kings College London, St Thomas’ Hospital, London, United Kingdom; 2 Center for Life Course Health Research, Faculty of Medicine, University of Oulu, Oulu, Finland; 3 Oulu University Hospital, Unit of Primary Care, Oulu, Finland; 4 MRC Lifecourse Epidemiology Unit, University of Southampton, Southampton General Hospital, Southampton, United Kingdom; 5 Seibersdorf Labor GmbH, Seibersdorf, Vienna, Austria; 6 Victoria University, Wellington, New Zealand; 7 NIHR Musculoskeletal Biomedical Research Unit, Nuffield Department of Orthopaedics, Rheumatology and Musculoskeletal Sciences, University of Oxford, Oxford, United Kingdom; 8 NIHR Nutrition Biomedical Research Centre, University of Southampton and University Hospital Southampton NHS Foundation Trust, Southampton General Hospital, Southampton, United Kingdom; 9 Department of Epidemiology and Biostatistics, MRC–PHE Centre for Environment & Health, School of Public Health, Imperial College London, London, United Kingdom; 10 Biocenter Oulu, University of Oulu, Oulu, Finland; 11 University of Nottingham, Nottingham, United Kingdom; University of Nottingham, UNITED KINGDOM

## Abstract

Several studies suggest that low birthweight resulting from restricted intrauterine growth can leave a metabolic footprint which may persist into adulthood. To investigate this, we performed metabolomic profiling on 5036 female twins, aged 18–80, with weight at birth information available from the TwinsUK cohort and performed independent replication in two additional cohorts. Out of 422 compounds tested, 25 metabolites associated with birthweight in these twins, replicated in 1951 men and women from the Hertfordshire Cohort Study (HCS, aged 66) and in 2391 men and women from the North Finland Birth 1986 cohort (NFBC, aged 16). We found distinct heterogeneity between sexes and, after adjusting for multiple tests and heterogeneity, two metabolites were reproducible overall (propionylcarnitine and 3-4-hydroxyphenyllactate). Testing women only, we found other metabolites associated with lower birthweight from the meta-analysis of the three cohorts (2-hydroxy-butyric acid and γ-glutamylleucine). Higher levels of all these metabolites can be linked to insulin resistance, oxidative stress or a dysfunction of energy metabolism, suggesting that low birthweight in both twins and singletons are having an impact on these pathways in adulthood.

## Introduction

The ways in which the foetus adapts to environmental factors including the changes during birth have an impact on the development of the foetus into adulthood and current research attempts to discern the mechanisms by which foetal programming affects adult disease [1]. As previously described [2] low birthweight (LBW), defined as weight at birth lower than 2500 grams, has been linked with childhood mortality [3], morbidity [4] and asthma [5]. LBW is also associated with multiple disorders progressing into adulthood, such as metabolic syndrome [6], Type 2 Diabetes [7], cardiovascular diseases [8], respiratory diseases [9] and osteoporosis [10].

Birthweight is directly affected by the length of gestation and prenatal growth rate but other factors such as maternal health, nutrition and smoking during pregnancy also play a role. Genetic contribution to birth weight has previously been investigated [11, 12], but to date, these variants only describe a small component of phenotypic variance. [11, 13]. Given the high relevance of LBW to health at older age, the molecular links between birthweight and age-related disease has attracted much interest.

DNA methylation differences in monozygotic (MZ) twins discordant for birthweight can control for many potential confounders, specifically differences in genetic background, early-life environmental exposures, age, sex and cohort effects. Although MZ twins do not necessarily share the same *in utero* environment, they are much more closely matched than unrelated individuals, and they offer a unique opportunity to study the link between early life factors and adult health. Currently, birthweight differences in twins are thought to originate from placental function and the differences in fetal access to nutrition that is affected by the position of the fetus *in utero* as well as the umbilical cord. Generally, singletons and twins develop at a similar rate until the 30th week of gestation, after which uterine restriction is a major contributing factor to twin births [14]. A lighter MZ twin has a greater likelihood of being genuinely growth restricted [15]. One recent study also suggested that discordance in birthweight in MZ twins is associated with differences in IQ and telomere length, which is a biomarker of ageing [16].

Several studies have looked at the interaction between birthweight and metabolomics. Wilson et al found that children at different stages of prematurity are metabolically distinct. Of 373,819 children born at term (>36 wk gestation), 26,483 near-term children (33–36 wk gestation), 4,354 very premature children (28–32 wk gestation), and 1,146 extremely premature children (<28 wk gestation), the amino acids showed consistent trends across categories of prematurity. The levels of arginine, leucine, and valine were at least 50% different between the cohorts of extremely premature and term children [17]. The Rhea cohort measured urinary metabolites at the end of the first trimester and found they are associated with increased risk of negative birth outcomes: preterm birth (PB) and fetal growth restriction (FGR) [18]. Another study collected urinary metabolites within 12 hours from 23 infants with intrauterine growth restriction (IUGR), large size for gestational age (LGA), and 10 control infants with appropriate weight for gestational age (AGA). The analyses suggest that increased urinary inositol may constitute a marker of altered glucose metabolism during fetal development in IUGR and LGA newborns [19]. Moreover, sex differences in the metabolites that correlate with birthweight are apparent in newborns [20]. More recently, Wurtz et al investigated the association between a panel of lipids, lipproteins and amino acids in 15414 adults and adolescents from Finland and 2874 adolescents from the UK and found that higher levels of cholesterol (except HDL), triglycerides and lipoproteins (except Apo A) were associated with lower weight at birth after adjustment for confounders [21].

In this study, we explored the hypothesis that birthweight differences are reflected in the metabolic profile and persist into adulthood and to do so we have used cohorts with very different age ranges, TwinsUK (age range 18–80 years) for discovery and two birth cohorts- one sampled at age 66 years and the other at 16 years for replication.

## Research design and methods

### Study cohorts

**TwinsUK** is the largest registry of adult twins in the United Kingdom. It started in 1992 and currently encompasses approximately 12,000 volunteer twins from all over the UK [22]. Not all the TwinsUK participants had birthweight data available and of those that did, not all had metabolite data so in total, we had 5,036 participants (1262 DZ pairs and 1257 MZ pairs) to study with regards birthweight. The study was approved by St. Thomas’ Hospital Research Ethics Committee, and all twins provided informed written consent. **The Hertfordshire Cohort Study**: from 1911 to 1948 in Hertfordshire (UK), each birth was notified by the attending midwife and the birthweight was recorded. Subsequently, health visitors who saw each child during infancy recorded his or her weight at the age of 1 year. 1,760 men and 1,447 women born in Hertfordshire between 1931 and 1939 who were singleton births and had both birth and infant weights recorded and were still resident in East Hertfordshire in the late 1990s were traced using National Health Service Central Registry data. Permission to contact 3,126 men and 2,973 women was obtained from the general practitioners. Of these subjects 1,684 (54%) of the men and 1,541 (52%) of the women agreed to take part in a home interview in which trained nurses collected information on the medical and social history. The subjects were then invited to a local clinic for several investigations [23, 24]. After sample preparation and validation, metabolite data was available for 1951 individuals, 850 males and 1101 females. The East and North Hertfordshire Ethical Committees granted ethical approval for the study and all participants gave written informed consent. **Northern Finland Birth Cohort 1986 (NFBC1986)** is a population-based birth cohort, including all mothers in the two northernmost provinces of Finland with children whose expected date of delivery was between July 1985 and June 1986. Birth data including birth weight were coded from hospital records at the time of delivery for 9432 live-born children. Blood serum samples were taken from 6265 children attending the clinical examination in the 16-year follow-up study between August 2001 and June 2002. In total, 2500 serum samples were sent for metabolite measurements; a random sample of 1644 and a selected set of 372 individuals exposed to gestational diabetes, gestational hypertensive disorders, preterm birth (<37 weeks) or on a high, (n = 242) or low (n = 242) tail of birth weight distribution. Accordingly, the analyses were adjusted for the sampling strata and gestational age. After sample preparation and validation, metabolite data was available for 2391 individuals, 1213 males and 1178 females. Ethical approval was obtained from the ethical committee of Northern Ostrobothnia Hospital District and all participants were given written informed consent.

### Metabolite selection

Two commercial panels of metabolites were used for discovery in the TwinsUK cohort. The correlation between the Biocrates and Metabolon panels, discussed below, was r = 0.678 (95% CI 0.45–0.87) Metabolites were collected from adults and analyses where birth weight information was available.

### Biocrates panel

The serum samples were collected after an overnight fast of all the study subjects. They were stored in −80°C freezers from which they were retrieved and sent to Germany for metabolite measurements. A targeted metabolomic assay was done in samples of fasting serum from participants in the British TwinsUK study (*n* = 1,003) using the Biocrates Absolute IDQ™-kit p150 (BIOCRATES Life Sciences AG, Innsbruck, Austria), as previously described [25, 26]. Briefly, the flow injection analysis (FIA) tandem mass spectrometry (MS/MS) method is used to quantify 163 known small molecule metabolites simultaneously by multiple reaction monitoring. Quantification of the metabolites is achieved by reference to appropriate internal standards. Reproducibility of the assay was performed in 23 serum samples. The mean of the coefficient of variation (CV) for the 163 metabolites was 0.07 and 90% of the metabolites had a CV of <0.10. Metabolite concentrations were normalized and scaled to have a mean of zero and a variance of 1.

### Metabolon panel

Metabolomics data in the TwinsUK samples was also measured by Metabolon Inc., Durham, USA as previously described [27]. Briefly, metabolite concentrations were measured in fasting blood samples using an untargeted GC/MS and LC/MS platform. Measurements were scaled by run-day median and inverse normalized. Missing data were imputed using the day minimum and metabolites with more than 20% missing were excluded from the analysis.

### Statistical analyses

All analyses were performed using R (version 3.1.2) using the lme4 (version 1.1) package.

Correlations between metabolites and birthweight were calculated using linear mixed models, correcting for age, BMI and family relatedness (as random intercept). The data was consistently normalized. The Biocrates and Metabolon platforms were tested in the TwinsUK cohort for associations with birthweight. Those showing an association with p<0.05, were carried forward for testing in the two additional cohorts (HCS and NFBC).

### Sample preparation

100μl of human serum were mixed with 10μl of internal standard. For protein precipitation, 850μl of methanol were added, the mixture was vortexed two times for 5 seconds and centrifuged for 3 minutes at 10°C and 14.000 rcf.

### LC-MS/MS-method 1 (metabolite measurement without derivatisation)

200μl of the supernatant were transferred into a new V-vial and dried under vacuum for 1 hour at 45°C. The dried material was re‐dissolved in 100μl of 20% methanol and 6 metabolites were measured directly using LC‐MS/MS. For direct analyses, an LC-MS/MS instrument of the TSQ Vantage series (Thermo Scientific), a reversed-phase C-18 column (Dionex Acclaim PolarAdvantage II, 4.6 x 50mm, 3μm) and following chromatographic conditions were used: 100% water/0.2% formic acid (1 min, isocratic), linear gradient to 100% methanol/0.1% formic acid (3 min), 100% methanol/0.1% formic acid (2.5 min, isocratic) and linear gradient back to 100% water/0.2% formic acid (1.5 min).

### LC-MS/MS-method 2 (metabolite measurement after derivatisation)

700μl of the supernatant obtained after protein precipitation and centrifugation were transferred into a second V-vial and dried under vacuum for 2 hours at 45°C. For derivatisation, 100μl of 3N HCl/1-butanol were added, the mixture was vortexed, incubated at 60°C for 7.5 minutes and dried under vacuum for 45 min at 45°C. The dried material was re‐dissolved in 100μl of 20% acetonitrile/0.1% formic acid and 19 metabolites were measured using LC-MS/MS. For analyses of derivatised samples, an LC‐MS/MS instrument of the TSQ Quantum Access Max series (Thermo Scientific), a reversed phase C‐8 column (Zorbax XDB, 4.6x75mm, 3.5μm) and following chromatographic conditions were used: 15% acetonitrile and 85% water (0.1 min), linear gradient to 100% acetonitrile (6.5 min), 100% acetonitrile (1.5 min, isocratic) and linear gradient back to 15% acetonitrile and 85% water (1 min).

### Validation

Each standard was measured three times and the *limits of quantitation and detection* (LOQs and LODs) were determined using ValiData3.00. To evaluate *matrix effects*, these standards were mixed with 100μl of human control serum from a healthy male volunteer and also analysed. No matrix interferences were observed. To guarantee *repeatability*, human control serum was analysed for 5 times on three different days. Repeatability was confirmed. To test the *precision* of the method, human control serum was measured ten times in a row. *Robustness* was tested by varying three steps during sample preparation, namely vortexing of samples (one time/30 seconds vs. two times/5 seconds), centrifugation (6 minutes instead of 3 minutes), and derivatisation time (10 minutes instead of 7.5 minutes). Each modification was analysed three times. Results showed that the methods were precise and robust.

Statistical analysis discovery and replication. Cohorts and sexes were analysed separately. Because the metabolon panel does not give absolute but only relative concentrations that are then normalized we also standardized the replication data so that every metabolite has a mean = 0 and a standard deviation of 1 in each of the cohorts. The results of the 25 metabolites carried out for replication were meta-analyzed using inverse variance fixed effect and meta-analysis implemented in the R package meta (version 4.3). In total 422 different compounds were tested at the discovery stage (the sum of known Metabolon + Biocrates is 455 compounds, but 23 metabolites are included in both panels). Therefore to adjust for multiple testing the Bonferroni p-value is 1.1 x 10^−4^

### ROC analysis

Relative (or receiver) operating characteristic (ROC) curves are a graphical representation of the relationship between sensitivity and specificity of a laboratory test over all possible diagnostic cutoff values [28].

## Results

The descriptive characteristics of the discovery and replication cohorts are presented in **Table 1.**

**Table 1:** Descriptive statistics of TwinsUK, Hertfordshire and North Finland birth cohorts.

cohort	TwinsUK	NFBC1986	NFBC1986	HCS	HCS
country	UK	Finland	Finland	UK	UK
type of study	Twins	singletons	singletons	singletons	singletons
sex	Females	Males	Females	Males	Females
n	5036	1213	1178	850	1101
birthweight kg (SD)	2.42 (0.58)	3.63 (0.72)	3.43 (0.71)	3.53 (0.52)	3.34 (0.49)
BMI kg/m2 (SD)	25.31 (4.68)	21.36 (3.78)	21.34 (3.31)	27.16 (3.81)	27.39 (4.72)
age years (SD)	54.01 (13.7)	16.04 (0.38)	16.05(0.38)	65.96 (2.81)	66.61 (2.71)

Twenty five metabolites from the Metabolon and Biocrates platforms were identified as correlated with birthweight in the TwinsUK cohort (**Table 2**). Assays were therefore developed to test these metabolites in serum from the two singleton replication cohorts from the UK and Finland comprising both men and women (**Table 1**).

**Table 2:** Metabolites associated with birthweight in the TwinsUK cohort.

METABOLITE	beta	SE	Pvalue	platform
Acetylcarnitine	-0.040	0.011	3.20E-04	Biocrates
Aspartate	-0.068	0.026	1.17E-02	Metabolon
Dodecanoylcarnitine	-0.039	0.012	7.40E-04	Metabolon
g-Glutamylleucine	-0.062	0.025	2.41E-02	Metabolon
g-Glutamylvaline	-0.071	0.026	1.09E-02	Metabolon
Glutamate	-0.053	0.024	3.41E-02	Metabolon
Glutarylcarnitine	-0.037	0.010	2.60E-04	Biocrates
Glycine	0.087	0.027	1.92E-03	Metabolon
C-glycosyltryptophan	-0.107	0.023	4.29E-06	Metabolon
Hexadecanedioic-acid	-0.021	0.011	4.96E-02	Metabolon
Isovalerylcarnitine	-0.067	0.025	1.04E-02	Metabolon
Kynurenine	-0.082	0.024	6.11E-04	Metabolon
2-methylbutyroylcarnitine	-0.080	0.026	5.18E-03	Metabolon
N-Acetylglycine	0.068	0.027	1.60E-02	Metabolon
N-Acetylthreonine	-0.078	0.026	1.79E-02	Metabolon
Oleoylcarnitine	-0.066	0.026	1.14E-02	Metabolon
Phenylacetylglutamine	-0.054	0.025	4.49E-02	Metabolon
Propionylcarnitine	-0.045	0.014	1.00E-03	Metabolon
Pyridoxic-acid	-0.042	0.025	4.34E-02	Metabolon
Succinylcarnitine	-0.102	0.026	3.91E-04	Metabolon
Tyrosine	-0.019	0.008	1.90E-02	Metabolon
Urea	-0.106	0.024	1.38E-05	Metabolon
Uric-acid	-0.099	0.024	3.86E-05	Metabolon
x2-hydroxy-butyric-acid	-0.094	0.026	3.06E-04	Metabolon
x3-4-hydroxyphenyllactate	-0.053	0.026	3.87E-02	Metabolon

Several metabolites show strong unadjusted associations in women, while we found limited evidence of an association between the metabolites tested and birthweight in men (**Table 3**).

**Table 3:** Metabolites associated with birthweight (adjusted for age and BMI) in both sexes from all 3 cohorts.

	HCS men		NFBC men		Meta analysis: men			HCS women		NFBC women		Meta analysis: women		
metabolite	B	SE	B	SE	B	SE	P-value	B	SE	B	SE	B	SE	P-value
2-hydroxy-butyric-acid	-0.009	0.018	0.114	0.033	0.004	0.016	0.78	-0.055	0.017	-0.054	0.035	**-0.0682**	**0.013**	**3.19E-07**
Propionylcarnitine	-0.037	0.018	-0.021	0.034	-0.041	0.016	0.0116	-0.029	0.020	-0.092	0.034	**-0.0468**	**0.011**	**2.26E-05**
Urea	0.000	0.018	-0.057	0.037	-0.012	0.017	0.46	-0.028	0.017	-0.054	0.039	-0.0570*	**0.013**	**2.05E-05**
2-methylbutyroylcarnitine	-0.024	0.018	-0.018	0.035	-0.034	0.016	0.0358	-0.030	0.017	-0.092	0.036	-0.0531*	**0.013**	**6.29E-05**
Acetylcarnitine	-0.019	0.018	0.081	0.038	-0.010	0.016	0.54	-0.022	0.017	-0.036	0.039	-0.0359*	**0.009**	**6.78E-05**
Isovalerylcarnitine	-0.010	0.021	-0.011	0.036	-0.012	0.019	0.50	-0.030	0.019	-0.107	0.036	-0.0552*	**0.014**	**0.00011**
γ-Glutamylleucine	-0.005	0.016	-0.008	0.020	-0.012	0.013	0.34	-0.029	0.025	-0.050	0.019	**-0.05478**	**0.014**	**0.00011**
Uric-acid	0.004	0.015	-0.021	0.034	-0.002	0.014	0.88	-0.031	0.022	-0.023	0.035	-0.05741	0.015	0.00015
Oleoylcarnitine	-0.019	0.016	0.030	0.025	-0.011	0.013	0.43	-0.045	0.019	-0.014	0.026	-0.05217	0.014	0.00013
Kynurenine	-0.002	0.019	-0.008	0.036	-0.012	0.017	0.49	-0.027	0.017	-0.037	0.037	-0.04838	0.013	0.00022
Glycine	0.004	0.018	0.020	0.035	0.006	0.016	0.72	0.026	0.015	0.102	0.038	0.0433	0.013	0.0006
3,4-hydroxyphenyllactate	-0.040	0.020	-0.009	0.036	-0.045	0.018	0.0135	-0.031	0.017	-0.058	0.037	-0.04641	0.014	0.00061
γ -Glutamylvaline	0.001	0.018	-0.004	0.019	-0.005	0.014	0.73	-0.013	0.016	-0.033	0.020	-0.03502	0.012	0.00313
C-glycosyltryptophan	-0.049	0.023	0.022	0.030	-0.033	0.019	0.095	-0.006	0.014	-0.011	0.032	-0.03758	0.011	0.0010
Succinylcarnitine	-0.034	0.019	-0.016	0.032	-0.038	0.017	0.0297	-0.005	0.018	-0.031	0.032	-0.04044	0.014	0.0037
Tyrosine	0.002	0.012	-0.016	0.032	-0.006	0.011	0.59	-0.023	0.027	-0.016	0.033	-0.0209	0.007	0.0055
Glutarylcarnitine	-0.003	0.019	-0.038	0.036	-0.008	0.017	0.65	0.028	0.015	-0.070	0.037	-0.0181	0.008	0.0251
Pyridoxic-acid	-0.036	0.018	-0.032	0.036	-0.036	0.016	0.0357	-0.001	0.015	-0.076	0.037	-0.0162	0.012	0.1945

(*) I^2^ >72% Cochrane’s Q < 0.0.05.

The random effect meta analysis summary statistics for these four compounds are as follows: Urea: beta (SE) = -0.0718 (0.0297) p = 0.016; 2-methylbutyroylcarnitine: beta (SE) = -0.0731 (0.0293) p = 0.0125; Acetylcarnitine: beta (SE) = -0.0731 (0.0293) p = 0.0125; Isovalerylcarnitine: beta (SE) = -0.0716 (0.0293) p = 0.0147

Overall, we found that there was heterogeneity in the associations between metabolites and birthweight in both males and females (**Table 4**)

**Table 4:** Meta-analysis of metabolites showing nominal evidence of association in both sexes (adjusted for age and BMI). Fixed effects (FE) summary effect sizes with standard errors are shown for all metabolites showing a p<0.05. Random effects (RE) for those where heterogeneity (I^2^) is higher than 40% or with a Cochrane’s Q < 0.10.

metabolite	FE: Beta	FE: SE	FE: p-value	I^2^	RE: Beta	RE: SE	RE: p-value
**Propionylcarnitine**	**-0.0452**	**0.009175**	**8.43E-07**	**0%**			
**2-methylbutyroylcarnitine**	**-0.0458**	**0.010312**	**9.42–06**	**62.1%**	**-0.060**	**0.019**	**0.001**
**3-4-hydroxyphenyllactate**	-0.0394	**0.010859**	**2.43–05**	**21.2%**			
2-hydroxy-butyric-acid	-0.0300	0.010369	0.00014	77.2%	-0.036	0.024	0.131
Urea	-0.0398	0.010556	0.00014	74.0%	-0.056	0.023	0.015
Acetylcarnitine	-0.0277	0.007923	0.00015	33.0%			
Isovalerylcarnitine	-0.0322	0.011408	0.00048	47.3%	-0.040	0.016	0.011
Glycine	-0.0353	0.010019	0.00328	61.9%	0.041	0.017	0.018
Uric-acid	-0.0320	0.010333	0.00729	68.9%	-0.046	0.020	0.021
γ-Glutamylleucine	-0.0228	0.00968	0.00088	36.6%			
Kynurenine	-0.0363	0.010514	0.00078	42.8%	-0.031	0.014	0.035
Oleoylcarnitine	-0.0394	0.009753	0.00104	55.4%	-0.023	0.014	0.109
γ-Glutamylvaline	-0.0165	0.009155	0.01260	38.8%			
C-glycosyltryptophan	-0.0231	0.009886	0.00023	77.7%	-0.032	0.022	0.149
Succinylcarnitine	**-0.0452**	0.010891	0.00029	58.2%	-0.037	0.017	0.029
Tyrosine	**-0.0457**	0.006343	0.00873	0%			
Glutarylcarnitine	**-0.0458**	0.007392	0.02603	75.9%	-0.018	0.018	0.319
Pyridoxic-acid	-0.0394	0.010079	0.0219	23.1%	-0.022	0.028	0.441

After adjusting for multiple tests two metabolites are associated in both sexes combined, namely propionylcarnitine and hydroxyphenyllactate (**Table 4**). A larger number of metabolites are associated in women including 2-hydroxy-butyric-acid, and acetylcarnitine (**Table 3**).

Given the heterogeneity between males and females and the lack of clear associations in men because of the lower sample size we concentrated on the metabolite markers in women. After excluding metabolites that showed high collinearity (by means of a stepwise regression) a logistic regression model was fitted using all metabolites that showed nominal association with birthweight in females. The outcome was low birthweight defined as the bottom tertile of the distribution in each cohort separately. We calculated the area under the curve (AUC) based on the coefficients from the meta-analysis. For TwinsUK the AUC was 0.592 with 95% confidence interval of 0.575–0.608; for HCS females it was 0.584 [0.547–0.621] and for NFBC females 0.606 [0.572–0.640].

## Discussion

In this study we report a clear metabolomic signature for lower birthweight detectable in adolescence, adult and old age in three independent cohorts looking at a vast array of metabolic pathways (not just lipids and lipoproteins).

We report that in women there are at least five metabolites consistently negatively associated with weight at birth. We find only two acylcarnitines associated with birthweight in men. This could be due to the study design which used a female only cohort for the discovery or it might reflect true differences between men and women which have been reported in newborns [20]. It is also worth noting that males weigh 136g more than females at term birth [29]. The reason for this is unclear and common theories include the action of androgens [30], sexual bias in metabolism [31] or that the Y-chromosome may be involved in ways that are yet to be elucidated [32].

Twins are an important group to study regarding birthweight because multiple births have been on the rise, with an increase of almost 50% from 1984–2004 in the United Kingdom (98.5% of these are twins), and they have high rates of complications (www.twinsuk.co.uk). The key assumption is that weight differences in twin pairs come from random differences in access to nutrition; for example, position in uterus/umbilical cord. Dizygotic twins (DZs) only share 50% of their genes (like siblings) and almost always have a separate placenta. It is believed that access to nutrients in DZ twins may differ due to mass/function of placenta or placental lesions but these effects are usually moderate. On the other hand, MZ twins are formed from the division of a single ovum, post-fertilization and can have one or two chorions. Severe differences in nutritional intake due to positioning and/or umbilical cord insertion can lead to greater weight discrepancies, and therefore a lighter MZ twin is likely to be genuinely growth-restricted [33]. Maternal behaviour such as smoking status and pregnancy risk factors may influence the weight at birth of a child and thus the twin model accounts for confounders related to mother and pregnancy because twins share the same uterine environment and have the same gestational age. Therefore, the observed effect of birthweight in twins comes from intra-uterine growth alone.

The most strongly associated metabolite associated in the twins, C- GlyTrp, is only modestly associated in the singleton cohorts (Table 3) and shows very strong heterogeneity. This compound is also highly correlated with chronological aging in both singletons and twins but the association with birthweight (already reported in Menni et al 2013) appears to be seen predominantly in twins [27].

The two compounds associated overall (in both sexes) are an acylcarnitine and hydroxyphenyllactate. Acylcarnitines are derived from mitochondrial acyl-CoA metabolism and have been associated with diet-induced insulin resistance [34]. Acylcarnitines comprise an acyl group esterified to L-carnitine, which enables them to cross the mitochondrial membrane. Several recent studies show increased levels of multiple acylcarnitines in obese, insulin resistant subjects [35] suggesting that accumulating acylcarnitines can interfere with insulin signalling. The negative correlation between birthweight and acylcarnitintes is consistent with the known role of birthweight in determining insulin resistance in later life [36]. The current data pinpoint a specific pathway that appears altered in adulthood in individuals with lower birthweight.

In infants high levels of hydroxyphenyllactate correlate with citrin deficiency [37]. Citrin is the hepatic mitochondrial aspartate-glutamate and its deficiency correlates with mitochondrial dysfunction. In adults, hydroxyphenyllactate is one of several markers for metabolic dysregulation in ovarian cancer [38]. In both instances hydroxyphenyllactate appears to be a marker for metabolic dysregulation and mitochondrial dysfunction.

A compound associated with birthweight in women after multiple replications is alpha hydroxybutyrate. Alpha-hydroxybutyric acid, is produced in mammalian tissues (principally hepatic) that catabolize L-threonine or synthesize glutathione. Oxidative stress or detoxification demands can dramatically increase the rate of hepatic glutathione synthesis [39]. Alpha-hydroxybutyrate has been shown is an early marker for both insulin resistance and impaired glucose regulation. The underlying biochemical mechanisms may involve increased lipid oxidation and oxidative stress [40]. This association with low birthweight, although seen only in women, is consistent with the idea that the biochemical pathways in adulthood altered by weight at birth, are linked to insulin resistance.

The final compound associated with low birthweight is γ-glutamylleucine. Gamma -glutamylated amino acids are established markers of oxidative stress and reflect glutathione turnover [41]. Under conditions of oxidative stress, intracellular glutathione turnover is increased which in turn activates the enzyme γ-glutamyl transpeptidase to cleave extracellular glutathione to release gamma-glutamylated amino acids which are taken up for intracellular glutathione reconstitution A drop in such amino acids has been reported in response to vitamin E treatment [41]. We find here that higher levels of γ-glutamyl amino acids are correlated in all three cohorts with lower birthweight, indicative of a higher oxidative stress status in adulthood.

Our results are consistent with a study in 3680 newborns that were metabolomically profiled for a panel of 12 aminoacids and 37 acylcarnitines. That study found that circulating levels both amino acids and acylcarnitines were correlated with birthweight females, whereas only levels of acylcarnitines were associated in males [20]. The two compounds that we find associated in both men and women here is propinylcarnitine, i.e. an acylcarnitine.

Having tested 422 distinct compounds from very diverse metabolic pathways for discovery, we have identified four types of compounds (acylcarnitines, gamma-glutamylleucine, hydroxyphenyllactate and alpha-hydroxybutyric acid) for which higher levels in adulthood correlate with lower weight at birth. Acylcarnitines and alpha-hydroxybutyric acid are directly linked to insulin resistance. Higher levels of gamma-glutamylleucine and alpha-hydroxybutyric acid are both associated to oxidative stress and hydroxyphenyllactate appears to be a biomarker of mitochondrial dysfunction. This is compatible with the DOHaD hypothesis but it also points to the critical role of lower insulin sensitivity and poor energy metabolism in linking deprivation in early life to poor health outcomes in later life.

### We note several study limitations

There is strong inter-study heterogeneity for some of the associations, which may be due to the very different age ranges in these studies, and some of it is introduced by the differences between sexes. Moreover, the results apply only to populations from Northern Europe (Finland and the UK). On the other hand, we have used three different designs, one of elderly individuals all from a local area (HCS), a birth cohort sampled at age 16 from Northern Finland, and a UK-wide twin cohort, hence the results should be generalisable to changes that are established in early development that remain throughout lifecourse. In addition, both UK cohorts and the Finnish study have been shown to be fairly well representative of the UK/ Finnish populations as described in the Cohort Profile papers [12, 31, 42].

Low birthweight has been associated, however, with clinical phenotypes that are not directly related to insulin resistance (e.g. osteoporosis). It is possible that the link with oxidative stress and energy metabolism underlies the adverse health outcomes indirectly. It is also likely that other metabolomic signatures in adulthood remain undetected with our approach. The results presented here suggest that investigating the longitudinal changes in the specific metabolites detected here may prove particularly useful in understanding the factors influencing unhealthy aging due to early exposures.

In conclusion, metabolomic profiling detects changes in adolescence, adulthood and old age that are linked to insulin resistance and oxidative stress, but there is clear heterogeneity between men and women.
